# Gestion optimisée des intrants pour une réponse efficace en contexte de pandémie au Cameroun

**DOI:** 10.4102/jphia.v16i1.858

**Published:** 2025-05-30

**Authors:** Joseph Fokam, Cyrille Alain Abega Abega, Ezechiel Ngoufack Jagni Semengue, Aissatou Abba, Désiré Takou, Grace Angong Beloumou, Sandrine Claire Djupsa Ndjeyep, Alex Durand Nka, Davy-Hyacinthe Anguechia Gouissi, Derrick Ayuk Ngwese Tambe, Chenwi Collins, Aude Christelle Ka’e, Michel Carlos Tommo Tchouaket, Rachel Audrey Nayang Mundo, Aurelie Minelle Kengni Ngueko, Naomi-Karell Etame, Larissa Gaelle F. Moko, Evariste Molimbou, Willy Leroi Togna Pabo, Pamela Patricia Tueguem, Nadine Fainguem, Lionele F. Mba, Laeticia Grace Yatchou, Nafissatou Ibnou, Thaddée Bienvenu Onana, Yap Boum, Alain Georges Etoundi Mballa, Alexis Ndjolo

**Affiliations:** 1 Chantal BIYA International Reference Centre for Research on HIV/AIDS Prevention and Management, Yaoundé, Cameroon; 2 National Committee for the Fight against AIDS, Ministry of Public Health, Yaoundé, Cameroon; 3 National Centre for the Coordination of Public Health Emergency Operations, Ministry of Public Health, Yaoundé, Cameroon; 4 Faculty of Health Sciences, University of Buea, Buea, Cameroon; 5 Faculty of Medicine and Biomedical Sciences, University of Yaoundé I, Yaoundé, Cameroon; 6 University of Rome Tor Vergata, Rome, Italy; 7 Faculty of Health Sciences, University of Buea, Buea, Cameroon, Belgium; 8 Faculty of Medicine and Health Sciences, University of Antwerp, Antwerp, Belgium; 9 National Public Health Laboratory, Ministry of Public Health, Yaoundé, Cameroon; 10 National Public Health Emergency Operations Coordination Centre, Ministry of Public Health, Yaoundé, Cameroon; 11 Institut Pasteur de Bangui, Bangui, République Centrafricaine; 12 Ministry of Public Health, National Public Health Emergency Operations Coordination Centre, Yaoundé, Cameroon

**Keywords:** COVID-19, stock supply, logistic management, CIRCB, Cameroon

## Abstract

**Optimised supplies management for an efficient response in a context of a pandemic in Cameroon**.

**Background:**

Response strategy against COVID-19 relies on adequate materials and consumables supply.

**Aim:**

As a national reference COVID-19 laboratory in Cameroon, the Chantal BIYA International Reference Center (CIRCB) has evaluated the consumption of materials and consumables supply for PCR, variant screening and sequencing, in view of better prediction of the needs.

**Setting:**

Chantal Biya International Reference Centre for research on human immunodeficiency virus (HIV) and acquired immunodeficiency syndrome (AIDS) prevention and management (CIRCB), Yaoundé-Cameroon.

**Methods:**

This was a descriptive cross-sectional study conducted between August 2021 and July 2022 which focused on stock supply evaluation through analysis of data from services performed at CIRCB, Cameroon. The weighted averages method was used in statistics.

**Results:**

Overall, 31 453 samples were received, 37 008 extractions and 37 248 PCRs were performed for COVID-19 diagnosis, representing an average monthly consumption of 3084 extractions and 3104 PCRs for ~2621 samples/month (~1.17 extractions/sample and ~1.18 PCRs/sample). Of the 2238 (7.1%) positive cases diagnosed, 265 were processed for variants’ screening and 200 to sequencing due to their high cycle threshold (CT < 25). In screening, 288 tests were used, i.e. 1.08 tests/sample. For sequencing, 279 tests were used, i.e. 1.39 tests/sample.

**Conclusion:**

The estimates obtained reveal a precise quantification of the supplies used for diagnosis, variant screening and sequencing during a season of high COVID-19 epidemics at CIRCB-Cameroon.

**Contribution:**

Adequate implementation of this logistical strategy would optimize supply management and help to save associated costs while ensuring effective genomic surveillance or response when faced with any public health incident in Cameroon.

## Introduction

Apparue dans la ville de Wuhan en Chine en décembre 2019, l’épidémie de coronavirus (COVID-19) a été déclarée comme pandémie mondiale le 11 mars 2020 par l’Organisation Mondiale de la Santé (OMS)^1,2,3,4^. L’Afrique, a connu son premier cas le 27 février 2020 au Nigéria^4^ et la maladie s’est peu à peu répandue, atteignant en quatre (04) ans plus de 9 546 789 cas confirmés et 175 421 décès^5^. Le Cameroun, qui occupe la deuxième position parmi les pays les plus touchés d’Afrique Subsaharienne après l’Afrique du sud, avait déclaré son premier cas le 06 mars 2020. En l’espace de quatre (04) mois seulement, le pays comptait déjà 12 270 cas, 313 décès et 7774 malades ayant recouvrés la guérison (à la date du 24 juin 2020)^4,6,7^.

Dans l’optique de limiter significativement la propagation de cette pandémie, plusieurs mesures de prévention avaient été mises en place par tous les gouvernements à l’instar de la distanciation sociale, le lavage régulier et systématique des mains, le port obligatoires des masques, la limitation du nombre de personnes dans les lieux publiques, la limitation sur le nombre de passagers dans les transports en commun, et bien d’autres^6,8^. Parallèlement, diverses stratégies avaient été développées par le ministère de la santé publique du Cameroun pour lutter contre la propagation de la COVID-19. Il s’agissait principalement de la stratégie de 3T (*Tracking Testing and Treatment*)^9^ ; l’adoption d’une stratégie de diagnostic moléculaire et la surveillance génomique de la COVID-19^4^. Ainsi, faisant suite à la synergie d’actions gouvernementales et de partenaires d’implémentation sur le plan international, les fournitures en réactifs, consommables et en équipements de protection (à l’instar des masques, des sur-blouses, des gants, des lunettes de protection etc…) ont permis d’équiper significativement les laboratoires choisis sur le plan national pour la riposte contre la COVID-19^4,6,8,9^. Toutefois, la gestion de ces intrants a principalement déterminé la réponse desdits laboratoires. Afin de gérer efficacement tous les cas incidents de COVID-19 et stopper la progression rapide de la maladie, l’implémentation d’une stratégie de gestion des approvisionnements en intrants serait capitale pour une réponse optimale du pays face à la COVID-19.

Notre objectif était d’évaluer, en vue de meilleures prévisions, les consommations en réactifs et consommables utilisés au laboratoire pour le diagnostic moléculaire et la surveillance génomique de la COVID-19 au Centre international de référence Chantal Biya pour la recherche sur la prévention et la prise en charge du Virus de l’Immunodéfiscience Humaine/Syndrome de l’Immuno Défiscience Acquise (VIH/SIDA), Yaoundé-Cameroun.

## Méthodes

### Type d’étude

Il s’agissait d’une étude rétrospective et transversale réalisée sur 12 mois (Août 2021 à Juillet 2022), portant sur le suivi des données de consommation des intrants pour le diagnostic moléculaire et la surveillance génomique de la COVID-19 au Cameroun.

### Contexte organisationnel

Nous avons mené cette étude au sein du laboratoire de virologie du Centre International de Référence Chantal Biya pour la recherche sur la prévention et la prise en charge du VIH/SIDA (CIRCB), Yaoundé-Cameroun. En tant que centre de référence COVID-19 pour le diagnostic et la surveillance génomique, le CIRCB est une institution gouvernementale du ministère de la santé publique du Cameroun qui est principalement porté vers la recherche sur la prévention et la prise en charge du VIH/SIDA. En outre, le CIRCB couvre : (a) le diagnostic précoce du VIH chez les nourrissons dans le cadre du programme national de prévention de la transmission mère-enfant (PTME); (b) le diagnostic des co-infections par le VIH ; (c) la mesure de la charge virale ; (d) la numération des lymphocytes T CD4 et CD8 ; (e) les tests biochimiques et hématologiques pour le suivi des médicaments ; (f) le test génotypique de résistance du VIH à des coûts subventionnés ; avec des programmes de contrôle de la qualité menés en partenariat avec *Quality Assessment and Standardization of Indicators* (QASI) et d’autres organisations internationales (http://www.circb.cm/btc_circb/web/).

### Collecte des données

Nous avons exploité les données collectées sur l’utilisation des réactifs et consommables pour les tests PCR (kits d’extraction et d’amplification), de criblage et de séquençage pendant la période d’étude.

## Extraction de l’acide nucléique, amplification et détection avec la plateforme manuelle : DaAn gene rRT-PCR

Pour le test DaAn gene reaction en chaine de la polymérase en temps réel [real-time reverse transcriptase polymerase chain reaction {rRT-PCR}], l’ARN viral a été extrait manuellement à partir d’un écouvillon nasopharyngé de 140 µL à l’aide du QIAamp Viral RNA Mini Kit (Qiagen Inc, Valencia, CA, United States [US]) conformément aux instructions du fabricant. L’amplification a été réalisée à l’aide du kit DaAn gene RT-PCR (www.daan-gene.com) sur le Quant Studio 5 (Thermofisher). Le protocole utilisait des sondes ciblant le gène du cadre de lecture ouvert (ORF1ab) et le gène de la protéine de la nucléocapside (N), avec une limite inférieure de détection de 500 copies/mL et une réaction d’amplification de 45 cycles. Conformément aux directives nationales du Cameroun, nous avons évalué la positivité du test Polymerase Chain Reaction (PCR) pour toute valeur du cycle seuil (CT) < 37^10^.

## Extraction de l’acide nucléique, amplification et détection avec la plateforme automatique : Abbott rRT-PCR

Pour le test Abbott rRT-PCR de détection du SARS-CoV-2, un aliquot de 1000 µL de chaque échantillon inactivé (500 µL d’écouvillon nasopharyngé + 500 µL d’eau exempte de DNase-RNase chauffée à 70°C pendant 10 minutes) a été chargé dans l’instrument Abbott *m2000sp*, combiné au master mix Abbott SARS-CoV-2 contenant un contrôle ARN interne, des amorces et des sondes ciblant à la fois le gène de l’ARN polymérase dépendant (RdRp), spécifique du SARS-CoV-2 ainsi que le gène de la protéine structurelle conservée de la nucléocapside (N) (www.fda.gov/media/136258/download).

L’amplification a été réalisée à l’aide du thermocycleur *m2000rt* après l’extraction automatisée et la préparation de l’échantillon à l’aide de l’instrument Abbott *m2000sp.* À la fin du processus d’amplification, les résultats négatifs ont été rendus comme ‘cible non détectée’ (aucune amplification n’a été observée après 37 cycles) ; tandis que les résultats positifs ont été rendus comme ‘cible détectée’ avec un nombre donné représentant le numéro de cycle (CN) auquel la phase de détection a été initiée. Cette valeur CN était inversement proportionnelle à la charge virale du patient. Selon le fabricant, la sensibilité de détection de ce test est de 100 copies/mL^10^.

### Procédure pour le criblage

Le criblage ou détection des variants préoccupants du SARS-CoV-2 via le génotypage par polymorphisme nucléotidique simple (*single nucleotide polymorphism*^10^ ; SNP) a été réalisée à l’aide du kit commercial SNPsig® SARS-CoV-2 (EscapePLEX CE) (PrimerDesign, UK), conformément au protocole du fabricant (PRIMER DESIGN, 2022).

### Procédure de séquençage

À la suite de l’expérience acquise dans le cadre du protocole de séquençage Sanger utilisé pour le génotypage du VIH^11,12,13^, le protocole de séquençage partiel du SARS-CoV-2 a été conçu à l’aide de nouvelles amorces spécifiques ciblant un fragment du génome du SARS-CoV-2 codant pour une partie de la protéine Spike.

### Variables

Les bons de livraisons et les registres de réceptions ont été exploités pour la collecte et le suivi des données de consommation des différents intrants pour le diagnostic moléculaire et la surveillance génomique de la COVID-19 au Cameroun. L’évaluation a porté essentiellement sur les kits d’extraction, d’amplification, de criblage, et de séquençage. Le niveau de service moyen (NSM) et la consommation moyenne mensuelle (CMM) de chaque intrant a été évaluée par la méthode des moyennes pondérées. L’inventaire ’des stocks a été effectué dans le but de comparer les stocks physiques aux stocks théoriques afin de ressortir les pertes et les avaries pour une meilleure prise de décision. Les Stocks Disponibles Utilisables (SDU) ont permis de déterminer le niveau d’utilisation des services en vue d’une meilleure planification.

Ainsi, la collecte de ces variables nous a permis de mettre en place un système d’information de gestion logistique (SIGL) dans ce laboratoire pour une manipulation facile des données.

### Analyse des données

Les données collectées ont été saisies sur Microsoft Excel 2021. Le logiciel IBM.SPSS^®^ Statistics V.20 a été utilisé pour les analyses statistiques.

### Considérations éthiques

Le Comité national d’éthique pour la recherche en santé humaine (CNERSH) a donné son accord pour que la présente étude n° 2022/01/1430/CE/CNERSH/SP) soit réalisée.

Étant donné que nous nous sommes limités à exploiter les données de consommation et de service déjà disponibles au niveau du laboratoire de virologie du CIRCB, aucune clairance éthique n’a été nécessaire. Toutefois, nous avons obtenu toutes les autorisations administratives avant le début de la collecte de données.

## Résultats

### Gestion des données de laboratoire

#### Diagnostic moléculaire de la COVID-19

Globalement, 31 453 échantillons ont été reçus et 36 144 tests ont été réalisés pour le diagnostic moléculaire de la COVID-19, soit un NSM de 2621 échantillons et 3012 tests pour une estimation de 1.15 tests/échantillon. En ce qui concerne

Concernant le diagnostic proprement dit, 2238 (7.1%) cas positifs ont été détectés (CT < 37). Le Tableau 1 présente l’ensemble des données de service et de consommation par mois sur l’ensemble de la période d’étude.

**TABLEAU 1:** Distribution des données de service et de consommation enregistrées chaque mois.

Données logistiques	Variables	Août 2021	Septembre 2021	Octobre 2021	Novembre 2021	Décembre 2021	Janvier 2022	Février 2022	Mars 2022	Avril 2022	Mai 2022	Juin 2022	Juillet 2022	Total
Données de services	Nbre d’Echantillons/mois	3427	4275	3298	2571	3236	3190	2954	2808	1822	1689	1333	850	31 453
	Nbre de tests patients réalisés†	3939	4914	3790	2954	3719	3666	3395	3228	2093	1940	1531	975	36 144
Données de consommation	Extractions‡	-	-	-	-	-	-	-	-	-	-	-	-	-
	Automatique	0	0	960	768	0	0	0	0	0	0	0	0	1728
	Manuelle	3998	4990	3159	2307	3774	3720	3444	3273	2120	1964	1548	983	35 280
	PCR§	-	-	-	-	-	-	-	-	-	-	-	-	-
	Automatique	0	0	960	768	0	0	0	0	0	0	0	0	1728
	Manuelle	4025	5021	3192	2334	3799	3745	3467	3294	2131	1974	1554	984	35 520

PCR, polymérase en temps réel [polymerase chain reaction].

† , Inclut aussi les tests repris pour confirmation de diagnostic;

‡ , Inclut les reprises et les contrôles de l’extraction;

§ , Inclut les reprises et les contrôles de la PCR.

**Donnees de criblage :** Parmi les cas positifs diagnostiqués, 265 ont été soumis au criblage sur la base de leur forte positivité à la PCR (CT < 25). Les données de consommations font état de trois (03) kits de 100 réactions utilisés et 288 tests réalisés soit une estimation de 1.08 test/échantillon.

**FIGURE 1:**
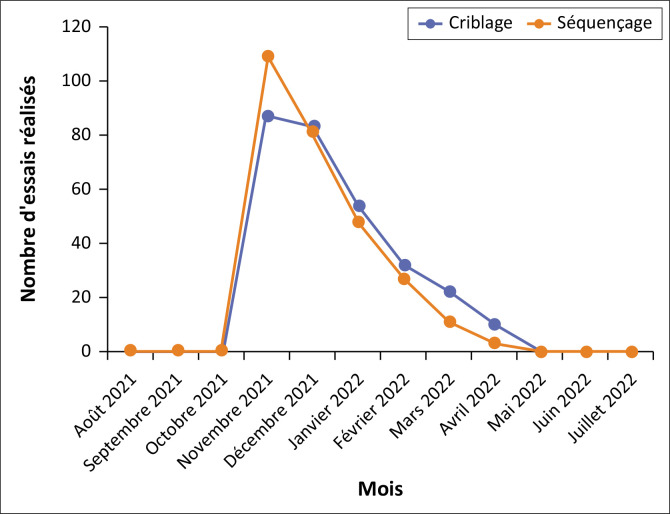
Suivi de la consommation en tests de criblage et séquençage.

**Données dé sequencage :** Pour le séquençage, 200 échantillons avec des charges virales élevés (CT < 25) ont été manipulés ; les données de consommation font état de six (06) kits de 48 réactions utilisés et 279 tests réalisés pour 130 séquences générées ; soit une estimation de 1.39 test/échantillon. La Figure 1 représente la distribution des tests effectués en criblage et en séquençage.

#### Inventaire des stocks

A la fin de notre période d’étude, le SDU en plateforme manuelle était de 40 kits d’extraction soit 1920 extractions avec 5 mois de validité. Quant à la plateforme automatique, le SDU était de 10 kits, soit 960 extractions dont la date de péremption était atteinte depuis déjà 3 mois.

En PCR, le SDU était de 22 kits d’amplification pour la plateforme manuelle soit 1056 réactions avec une validité de 4 mois. Quant à la plateforme automatique, le SDU était de 110 kits, soit 10 560 réactions avec encore 7 mois de validité.

En criblage, le SDU était de zéro kit tandis qu’en séquençage, il était de deux (02) kits, soit 96 réactions avec encore 7 mois de validité.

### Estimation des previsions necessaires d’intrants

Les données de quantifications obtenues à la fin de cette période d’étude ont permis d’établir des prévisions nécessaires en intrants pour les extractions, la PCR, le criblage et le séquençage. Ainsi pour une meilleure estimation des besoins réels, nous pouvons proposer la formule ci-après :


B=N*(C+0.2) [Eqn 1]

où : B = le besoin estimatif réel;

N = le nombre moyen de patients attendus sur une période donnée;

C = coefficient de quantification (qui varie dans cette étude en fonction que l’on recherche le besoin nécessaire pour l’extraction, la PCR, le criblage ou le séquençage)

NB : 0.2 représente le facteur de multiplication de perte^14^.

Ainsi, en supposant un effectif moyen de 2621 patients parmi lesquels environ 190 sont positifs et ont de fortes virémies et en considérant les valeurs de 1.17 ; 1.18 ; 1.08 ; et 1.39 comme coefficients de quantification pour les besoins respectifs en extractions, PCR, criblage et séquençage, nous obtenons les estimations ci-après :

- Be = 2621 * (1.17 + 0.2) = 3591 extractions nécessaires, soit ~75 kits de 48reactions.
- Bp = 2621 * (1.18 + 0.2) = 3617 PCR nécessaires, soit ~76 kits de 48 réactions.
- Bc = 190 * (1.08 + 0.2) = 243 tests de criblage nécessaires, soit ~3 kits 100 réactions.
- Bs = 190 * (1.39 + 0.2) = 302 tests de séquençage nécessaires, soit ~7 kits de 48 réactions.

## Discussion

Une quantification précise dans un laboratoire de santé est la garantie de la disponibilité des consommables et des réactifs en temps réel^15,16,17^. Elle permet de résoudre les problèmes de sur-stockage, de rupture de stock, du gaspillage de matériels et des ressources financières, en tenant compte du coût, du volume de matériel, de l’espace disponible, et de la variation du niveau de service du site^15,16^. La quantification dans un laboratoire permettra également une meilleure planification, une meilleure prise des décisions et aussi permettra une meilleure estimation des exigences dans les limites du budget disponible pour la réponse sanitaire^15,16,17^. Pour rappel, l’objectif de ce travail était d’évaluer, en vue de meilleures prévisions, les consommations en réactifs et consommables utilisés au laboratoire de virologie du CIRCB, dans le cadre du diagnostic moléculaire et la surveillance génomique de la COVID-19 au Cameroun.

Les données de consommation font état d’une surutilisation de la plateforme manuelle au dépend de l’automatique pour l’extraction et la PCR. Cette situation a conduit à des sous-stocks d’intrants pour la plateforme manuelle et des surstocks en intrants pour la plateforme automatique, dont certains ont atteint la date d’expiration sans être utilisés. Ceci pouvait se justifier par la durée de manipulation en plateforme automatique, qui était beaucoup plus longue que celle de la plateforme manuelle, d’où la préférence de cette dernière par rapport à l’automatique pour gérer l’état d’alerte en période de haute épidémie^7,18^. En ce qui concerne le criblage, les données de consommation soulignent un faible niveau de service, qui se justifie par le fait que la surveillance génomique n’est recommandée que pour les patients diagnostiqués positifs avec une forte virémie^10,19,20^. Rappelons que l’objectif du criblage était de détecter, parmi les patients positifs, ceux porteurs d’un variant préoccupant^21^. Ainsi, seuls les cas avec une symptomatologie atypique et une virémie élevée étaient sélectionnés pour criblage et le séquençage^10,19,20^. Ceci permettait donc en cas de positivité, d’activer les mécanismes pour le *contact tracing* dans les plus brefs délais^9^. Toutefois, la mise à disposition tardive des réactifs et le faible approvisionnement a conduit à une rupture rapide des intrants pour le criblage (Figure 1)^21^. Les données de consommations pour le séquençage ont révélé, comme dans le cas précédent, un faible niveau de service, le séquençage étant aussi une activité de surveillance génomique. Cependant, contrairement au criblage, l’inventaire a révélé un sous-stock en réactif de séquençage. Ceci pouvait s’expliquer par le fait que la technique a été empruntée des activités de surveillance de la résistance du VIH déjà effectués dans le laboratoire^11,12,13^. Un point important à noter est que nous avons toutefois adressé une demande de restitution du matériel à la hiérarchie (ici le *Ministère de la Santé Publique du Cameroun*), pour une redistribution dans les autres laboratoires agréés des réactifs et consommables non encore avariés mais en surstocks dans nos magasins ; ceci dans l’optique d’une meilleure gestion des ressources disponibles sur le plan national.

En outre, deux facteurs incontournables dans la mise en place d’une stratégie de gestion logistique, telle que décrite dans ce travail, sont l’équipement et la ressource humaine. En effet, les équipements dans un laboratoire conditionnent le type et même la quantité d’intrants nécessaires pour un niveau de service satisfaisant, spécialement en contexte d’urgence sanitaire. Lorsque l’on dispose donc d’un personnel suffisamment motivé dans le travail et formé à l’utilisation de ces équipements, promptitude, justesse, fiabilité et précision ne peuvent que découler du service rendu, pour un plus grand impact/bénéfice perçu dans la communauté.

## Conclusion

Il ressort donc au terme de ce travail, que l’intégration des outils logistiques dans la gestion des intrants en fonction des données de services réalisés (nombre d’échantillons/patients reçus et nombre de tests) permettrait d’estimer de manière plus systématique les besoins réels, ce qui serait un atout dans la préparation d’une riposte ou en cas d’une urgence sanitaire telle que COVID-19. Compte tenu qu’une rupture de stocks ou un sous-stock en matériel peut rompre totalement le maillon d’une chaine d’activité dans un laboratoire en situation d’urgence, une gestion rationnelle des achats sur la base de quantification précise, permettrait de faire des économies budgétaires tout en assurant un approvisionnement constant des réactifs et les.
